# Psychiatric disorders in children attending a Nigerian primary care unit: functional impairment and risk factors

**DOI:** 10.1186/1753-2000-6-28

**Published:** 2012-07-31

**Authors:** Mosunmola Tunde-Ayinmode, Olushola Adegunloye, Babatunde Ayinmode, Olatunji Abiodun

**Affiliations:** 1 Department of Behavioral Sciences, University of Ilorin Teaching Hospital, Ilorin, Nigeria; 2 Department of Family Medicine/GOPD, University of Ilorin Teaching Hospital, Ilorin, Nigeria

**Keywords:** Psychiatric disorders, Functional impairment and risk factors, Primary care children, Nigeria

## Abstract

**Background:**

There is dearth of data on the level of functional impairment and risk factors for psychiatric morbidity in children attending primary care services in developing countries like Nigeria. The risk factors for psychiatric morbidity and functional impairment in children attending the primary care unit of a teaching hospital in Ilorin, Nigeria was therefore investigated to obtain data that could be used in improving service provision by primary care physicians.

**Methods:**

A cross-sectional two-stage design was employed for the study. The first stage involved administration of the Child Behavior Questionnaire (CBQ) to 350 children while the children’s version of the schedule for affective disorders and schizophrenia was used for the second stage involving 157 children, all high scorers on CBQ (score of ≥ 7) and 30% of low scorers (score < 7). Diagnosis of psychiatric disorders was based on DSM-IV criteria.

In addition, the Children Global Assessment Scale was used to assess the functional status of the children (score of ≤ 70 indicates functional impairment) while the mothers’ mental health status was assessed with the 12-item version of the General Health Questionnaire, a score of 3 or more on this instrument indicate presence of mental morbidity.

**Results:**

It was observed that 11.4% of the children had diagnosable psychiatric disorders and 7.1% were functionally impaired; and those with psychiatric disorders were more functionally impaired than those without. Thus, significant negative correlation was noted between CBQ scores and CGAS (r = 0.53; p < 0.001). Following logistic regression, younger age of children, frequent hospital attendance and maternal parenting distress independently predicted psychiatric morbidity while child psychopathology and maternal parenting distress predicted functional impairment.

**Conclusions:**

Child psychiatric disorders are prevalent in the primary care unit studied. Many of the risk factors identified in the study population are modifiable. Collaborative efforts between psychiatrists and primary care physicians could therefore help to reduce level of risk and functional impairment and psychiatric morbidity among children attending the primary care unit studied. It could also help improve referral rates of difficult cases to the child and adolescent psychiatric unit of the hospital.

## Background

Risk factors increase the chances of onset of psychiatric disorders, and when already present, of its worsening and perpetuation or chronicity. Protective factors such as, high self esteem, problem-solving and social skills, positive thinking, good physical health, educational opportunities and positive parenting and availability of social support systems, as examples are capable of modifying individual response to psychosocial stress [1,2]. The presence of multiple risk factors and absence of protective factors interacting are suggested as influential in psychopathology [1,2]. Mental health promotion and ill health prevention is anchored on this principle. Therefore identifying risk factors in childhood mental health has clinic and public health benefits. Knowing the most important risk factors may not only increase the frequency of detection at the clinic level but may serve preventive purpose in public health especially where they are modifiable [1].

Although prevalence of diagnosable child psychopathology in primary care varies widely globally, depending mainly on geographical and methodological factors [3-5]. In low resource countries an average of 14.3% has been estimated [5] and the commonest problems are anxiety disorders; depressive disorders; conduct disorders and delinquency; learning disabilities and mental retardation [4-6]; problems like ADHD and autistic spectrum disorders are not as commonly reported as in high income countries [7,8]. Considering this trend and the low rate of detection and treatment at the primary care level psychosocial risk factors for psychiatric morbidity in children remains a major area of research.

There is diversity in occurrence and character of risk factors as suggested by various studies [3,4,6,9] depending on biological, psychological and socioeconomic attributes and circumstances of the children. The factors include among others: chronic physical illness; frequent hospital attendance, younger age, not schooling, poor academic performance, physical and sexual abuse, gender, large family size, socioeconomic deprivations, adverse life and chronic life difficulties and parental loss [4,6,10-12]. Additional risk factors related to parents include: parental loss, parental low educational status, unemployment, marital problems; domestic violence, mental disorders and family dysfunction.

Unfavourable family environment is one of the most important negative contributors to children mental health [6]. The frequency of these mental health problems increases significantly when very many risk factors are present at the same time [2]. Conversely, when individual, family and social resources are robust then there is a reduced occurrence of mental health problems, particularly in children with fewer risk factors [2,6].

Functional impairment describes the impact of psychopathology on the life of the child with respect to daily life activities [13]. Functional impairment if undetected or unmanaged may affect the treatment and course of the psychiatric disorders; persistent functional impairment could also affect psychosocial development and eventually cause serious psychosocial burden in adult life [3,13,14]. Many studies have been devoted to investigating the relationship between psychopathology and functional impairment [3,15]. Interest in this area is because these variables may determine the need for special approach to the management of affected children [3]. In addition, characterization of these variables is important in case definitions, treatment efficacy assessment and as indicators of outcome [14]. Furthermore, functional impairment should be assessed routinely because improving the patient’s level of functioning is always an important goal of treatment.

Our child and adolescent psychiatric unit was recently established and encountering low patronage despite sensitization efforts. As part of effort towards service improvement we decided to study the risk factors of psychopathology and functional impairment in children at the general outpatient department (GOPD) of our hospital because it is a major source of referral to the psychiatry clinic. Apart from sensitizing primary care physicians at the GOPD, the knowledge of identified risk factors if exploited could potentially aid early detection and appropriate referral of cases.

So far, very few studies currently exist in Nigeria on risk factors and functional impairment in child psychiatric disorders. To the best of our knowledge none had been done in the North central region of Nigeria where our institution is located.

## Materials and methods

This study was conducted at the General outpatient department (GOPD) of University of Ilorin Teaching Hospital (UITH), Ilorin, Nigeria. The UITH is one of the 45 federally owned tertiary hospitals in Nigeria; it is a 445 bed hospital which has two 35 bed rural based secondary comprehensive health centres annexed to it. The UITH is located in Ilorin, Kwara state, North-central Nigeria; and has over 19 clinical departments offering specialist services to its host and 5 contiguous states. Ilorin is a cosmopolitan city with diverse culture and people but the indigenous people are predominantly Yoruba language speaking and Muslims. The GOPD is a walk-in unit of the hospital offering primary care services to all patients both young and old. The GOPD had 6 consultants and 17 resident doctors at the time of this study. Of these, 2 consultants and 6 resident doctors ran the pediatric and school clinics where the study took place. Children are also seen in the Pediatric department which offers both inpatient and outpatient specialist services which along with the GOPD provide most of the patients seen at our 5 year old child and adolescent psychiatric clinic. In Nigeria, majority of child mental health problems present to the primary care and the school health services which are still underdeveloped in terms of detecting, treating and providing health education. The same problems affect the secondary and tertiary levels of health care albeit to a lesser degree.

The study involved a two-stage cross sectional investigation of children aged 7-14 years and their mothers attending the GOPD over a period of 6 months. In the first stage, all consecutive clinic attendees during the study period were requested to participate in the study until the target sample size was attained. Children who were either too ill to take part or were unaccompanied by their mothers were excluded. The calculated sample size was 246; it was derived on the basis of a desired accuracy of 0.05 or 5% and confidence limits of 95% (Z score 1.96) and upper limit prevalence of child psychiatric disorder (primary outcome variable) in the target population of 20% (from a previous local study). n = (Z^2^)(p) (1-p)/d^2^ (z = confidence interval limits; n = sample size; p = known prevalence; d = degree of accuracy) [16]. It was however increased to 350 to take care of other secondary outcome variables.

Every consenting mother completed the socio-demographic data sheet designed by the authors. This consisted of two sections. The first section obtained information on the children (e.g. educational and developmental indices, medical history, consultation pattern in the preceding 6 months, family and parenting characteristics, etc.).

The second section gathered information on their parents (e.g. marital/occupational status; medical and psychiatric morbidities, etc.). The mothers also completed the parent version of the Child Behavior Questionnaire (CBQ).

Child Behavior Questionnaire (CBQ) [17] has 31- items, each item being rated from 0–2 thus producing a total score within the range of 0–62. In the present study a cut off score of 7 for CBQ was used as suggested in an earlier validation study done among children aged 7-14 years in a Nigerian population [18].

The mental health of each mother was assessed with the 12-item version of the General Health Questionnaire (GHQ-12) [19]. A validation study by one of us had earlier found the optimum cut-off point for GHQ-12 to be a score of 3 [19,20]. Mothers who were illiterates had the Yoruba version of the above questionnaires (produced through the process of back translation) read out to them by trained research assistants and their responses recorded. In all, 350 mothers and children participated in the first stage of the study while an additional 9 mothers refused participation for reasons of lack of interest and/or time, thus response rate was 97.5%.

The second stage assessment was conducted using the children’s version of the schedule for affective disorders and schizophrenia, present and life version (K-SADS-PL) [21]. This is a semi-structured diagnostic interview instrument designed to assess current and past episodes of psychopathology in children and adolescents in accordance with both DSM IIIR and DSM IV criteria.

The K-SADS-PL was administered by first interviewing the mother about her child’s symptoms, then the child was interviewed and a summary rating of each symptom based on the two sources of information was made [21].

Three trained senior residents in psychiatry without the knowledge of the first stage score administered the K-SADS-PL. Before commencement of study an inter rater exercise assessing the doctors on the instrument was conducted revealed a simple percentage agreement of about 93% for all diagnosable conditions on K-SADS. A total of 157 children and their mothers participated in the second stage assessment. This was made up of all those scoring ≥7 on CBQ (designated as high scorers) and 30% of those scoring <7 (designated as low scorers) selected by systematic random sampling of 1 in every 3 low scorers. All the mothers and their children approached for the second stage interview consented and were successfully interviewed. DSM-IV diagnosis was assigned as appropriate after each assessment.

The interviewers also completed the Children Global Assessment Scale (CGAS) with information obtained from the mothers. The CGAS is an instrument designed to measure functional impairment in children and has acceptable and discriminant validity [22,23] CGAS is rated on a 100-point score and a score of ≤70 indicates presence of functional impairment.

### Data analysis

Data analyses was done using EPI info version (6.02) [24] and SPSS version 15 for windows [25]. Statistical significance was set at p < 0.05. Further quality control was ensured during data score computations, coding and entering as it was done by only one person. All the risk factors that were significantly associated with child psychiatric disorders and functional impairment (independent variables) were subjected to multicolinearity check in the SPSS program before being entered into stepwise multiple regression analysis with backward elimination. Highly inter-correlated variables with statistically significant correlation coefficient greater than 0.9 in the correlation matrix were removed from the model. The program also created dummy or indicator variables for categorical variables that were not initially dichotomous. This method calculated the log odds ratio for each independent variable in the equation and generated the best fitting model after adjusting for others. The Hosmer - Lemeshow test which assesses the predictive accuracy of the model is generated by SPSS formed part of the assessment of the best-fitting model [25].

## Results and discussion

### Basic social data

The mean age of the 350 children was 9.75 ± 2.11. Boys constituted 51.7% while girls made up 48.3% of the study population. Majority of the children were in primary school (70.9%); 26.9% were in secondary school while 2.3% were not in school. Majority (over 90%) were living with their parents.

### Prevalence and risk factors for psychiatric morbidity

Forty children out of the 157 who had second stage assessment had psychiatric morbidity giving an overall prevalence rate of 11.4% on the basis of the study population. Enuresis was diagnosed in 21 (13.4%); conduct disorder in 6(3.8%); Attention deficit hyperactivity disorder in 5(3.2%) children; anxiety disorders in 4(2.5) children; depression in 2 (1.3%) and mental retardation in 2(1.3%) children.

With univariate analysis, presence of psychiatric morbidity in the children was found to be significantly associated with being younger (*X*^2^ = 4.76; p < 0.029); not attending (*X*^2^ = 13.43; p < 0.000) or performing poorly in school (*X*^2^ = 8.70; p < 0.003); having a chronic physical illness (*X*^2^ = 4.28; p < 0.039) and frequent hospital visits (*X*^2^ = 11.54; p < 0.009); having a mother who was experiencing parenting distress with one or more children (*X*^2^ = 14.80; p < 0.000) and also having a mother with mental ill-health (*X*^2^ = 3.49; p < 0.040) (Table 1).

**Table 1:** Risk factors for psychiatric morbidity in children

**Variables**	**Cases n (%)**	**Non-Cases n (%)**	**Chi Square**	**P value**
Age group in years (N = 157) 7–10 11-14	31(32) 9(15)	66(68) 51(85)	4.76	0.029
Educational status (N = 157) No formal education Primary school Secondary school	3(100) 33(28) 4(11)	0(0) 84(72) 33(89)	13.43	0.000
*School performance (N = 152) Good Poor	28(20) 7(64)	113(80) 4(36)	8.70	0.003
Presence of chronic physical illness (N = 157) Present Absent	11(44) 29(22)	14(56) 103(78)	4.28	0.039
Number of hospital visits in the last 6 months (N = 157) None One Two ≥ Three	8(24) 15(24) 3(9) 14(47)	25(76) 47(76) 29(91) 16(53)	11.54	0.009
Parenting distress with one or more children (N = 157) Present Not Present	13(62) 27(20)	8(38) 109(80)	14.80	0.000
Presence of psychological disorder In the mother by GHQ-12 (N = 157) Present Not Present	8(47) 32(23)	9(53) 108(77)	3.49	0.040

* 5 children were not attending school (3 never attended and 2 were withdrawn from primary school before study commenced).

n/N % in row brackets to the nearest whole number.

In the multiple logistic regressions of child psychopathology on independent variables (identified risk factors), multicolinearity check eliminated education and school performance from the model; subsequent regression produced three variables, younger age of the child, frequent hospital attendance and mothers’ experience of parenting distress as significantly associated with presence of psychiatric morbidity and therefore its best predictors (Table 2).

**Table 2:** Risk factors independently associated with psychiatric morbidity in children as confirmed by multiple logistic regressions

**Variables**		**Log odds ratio**	**95% confidence interval for log odds ratio**	**Level of significance**
Age group in years (N = 157)	11-14 7-10	2.657	1.084-6.513	0.033
Presence of chronic physical illness (N = 157)	Yes No	0.750	0.211-1.905	0.417
Frequency of hospital visits in last 6 months	≤2 >2	2.775	1.117-6.893	0.028
Maternal parenting distress with one or more children (N = 157)	Yes No	5.817	2.080-16.204	0.001
Presence of probable psychological disorder in the mother by GHQ-12 (N = 157)	Yes No	2.095	0.652-6.731	0.214

### Functional impairment

Based on the cut-off score of ≤ 70% for functional impairment, 25 (7.1%) out of 350 children were considered to be functionally impaired. Children with psychopathology were significantly more likely to be impaired on both CBQ and K-SADS than those without (p = 0.000). There was significant negative correlation between CBQ scores and CGAS (r = 0.53; p < 0.001) suggesting that the higher the probability of psychological disorders in the children the lower the level of functioning. However, children with enuresis were less likely to be functionally impaired (2/21; 9.5%) compared with other psychiatric diagnosis (4/19; 21.1%) (p = 0.000). Children who were functionally impaired were observed to be more likely to have developmental delays (*X*^2^ = 7.01; p < 0.013) and more likely to have chronic medical illness (*X*^2^ = 15.19; p < 0.000). Mothers of functionally impaired children were more likely to be experiencing parenting distress (*X*^2^ = 46.01; p < 0.000); more likely to have poor relationship with their husbands (*X*^2^ = 7.79; p < 0.005); and also more likely to experience poor support for child care (*X*^2^ = 3.43; p < 0.048). In addition these mothers were likely to be identified as having mental ill-health on GHQ-12 (Table 3). In the multiple logistic regressions of functional impairment on the independent variables (identified risk factors), two variables, presence of child psychopathology (Log odds ratio =10.67; p = 0.000; 95% confidence interval (CI): 2.93-38.92) and mothers’ experience of parenting distress (Log odds ratio =7.27; p = 0.005; 95% CI: 1.81-29.20) remained significantly associated with functional impairment at the maximum number of steps; and therefore best predictors of functional impairment.

**Table 3:** Factors associated with functional impairment in children

**Variables**	**Functionally impaired n (%)**	**Not functionally impaired n (%)**	**Chi square**	**P value**
Developmental delay (350)	Present	4(29) 10(71)	7.01	0.013
	Not Present	21(6) 315(94)		
Chronic physical (N = 350)	Present	10(22) 35(78)	15.19	0.000
	Not Present	15(5) 290(95)		
Child psychopathology (DSM IV) (N = 350)	Present	17(43) 23(57)	99.50	0.000*
	Absent	4 (1) 306(99)		
Mothers’ relationship with husband (N = 330)	Poor	3(38) 5(62)	7.79	0.005
	Good	19(6) 303(94)		
Husband’ support for mother in child care (N = 330)	Inadequate	3(23) 10(77)	3.43	0.048
	Adequate	19(6) 298(94)		
Maternal parenting distress (N = 350)	Present	12(39) 19(61)	46.01	0.000
	Not Present	13(4) 306(96)		
Mothers GHQ Score (N = 350)	≥3	8(29) 20(71)	17.70	0.000*
	<3	17(5) 305(95)		
Children’s CBQ Score (N = 350)	≥7	18(45) 22(55)	91.25	0.000*
	<7	7(2) 303(98)		

* = Fishers exact test.

n/N % in row brackets to the nearest whole number.

**Figure 1:**
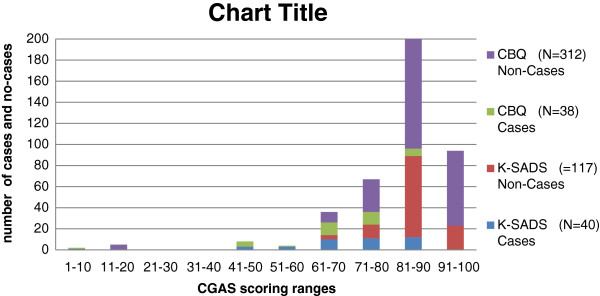
Distribution of Children Global Assessment Functioning Scores among Cases and Non-Cases as determined by K-SADS and CBQ.

Figure 1 is stacked bar charts of the distribution of CGAS scores (X axis) plotted against number of cases and non-cases on CBQ and K-SADS (Y axis). It suggests an over representation of children without psychological problems (non-cases) in the normal range of functioning and those with problems (cases) in the impairment range. It also shows presence of functional impairment without psychological morbidity (‘non-caseness’) in a few children and functional normality with psychological morbidity (‘caseness’) in a few others.

Our study has provided evidence that many children attending primary care services have DSM IV diagnosable psychopathologies. And that these impair their functioning in various domains of their daily life as indicated by low CGAS scores. Also there was significant correlation between presence of psychopathology and functional impairment. Children with severe psychiatric disorders constituted majority of those who had functional impairment. Educational, medical, developmental and family risk factors significantly influenced psychiatric morbidity and functional status. Multiple logistic regression analysis of risk factors provided evidence that younger age of children, frequent hospital visits and maternal parenting distress were the strongest predictors of psychopathology in our center and these factors also had significant association with functional impartment in the children. In addition, multiple logistic regressions of functional impairment also found presence of child psychopathology and of parenting distress as its best predictors. By and large evidence above suggests that common factors may indeed influence and predict psychopathology and functional impairment and this knowledge should be used in mental health promotion and illness prevention.

The overall prevalence of diagnosable psychopathology in this study (11.4%) was higher than that of functional impairment (7.1%). One study reported 8% prevalence rate of psychopathology in an outpatient population of adolescents [3]. In that study, all the patients investigated had functional impairment irrespective of psychopathology but those with psychiatric morbidity were differentially more impaired in terms of severity. In our study, of the 40 children with psychiatric morbidity 42.5% had functional impairment but 32% of the 25 children with functional impairment had no psychopathology. This latter subgroup of children were probably functionally impaired for other reasons such as chronic medical conditions or presence of individual or maternal psychosocial stressors or psychopathology that was subclinical as at the time of the study [3]. On the other hand 57.5% of the children had psychiatric diagnosis but no impairment. It may be that they were probably well adjusted or coping with their problems, the moderating role of the milder illness severity [26] cannot be ruled out since majority of them (82.3%) had the least stressful or severe condition (enuresis).

The majority of the children in our study with functional impairment were those with the more severe psychopathologies (e.g. mental retardation, ADHD, etc.), suggesting that severity of the disorder was a major factor that influenced impairment. For example, enuresis which was the mildest of the problems and it had impairment rate of 9.5% while all the children with ADHD, depression and mental retardation were all functionally impaired. Although a lot of parents are often worried about enuresis our study shows that majority did not have functional impairment this should help reassure parents about this condition.

The status of enuresis as the most frequent diagnosable psychopathology in this study agrees with a previous study in Ethiopia [27]. This urban community-based study with 5000 participants had enuresis as its primary research outcome variable and reported a prevalence of 12.3%. It also linked enuresis with increased risk of other DSM IV diagnoses. In the present study, prevalence of enuresis was 13.4% but contrary to suggestions from the Ethiopian study [27] enuresis was not associated with as serious psychological impairment judging by comparatively low rate of functional impairment (9.5%) found among affected children. The differences between the two studies may be linked to contextual socio-cultural and methodological factors. Although both studies are urban based, ours is hospital based, with smaller sample size and all diagnosable psychopathology as its primary research outcome variable not enuresis specifically.

Literature suggests that there are many risk factors for psychiatric morbidity and functional impairment in children [9,28]. Several studies have focused on predictors of functional impairment and the association to psychopathologic risks factors [9,28-30]. Review of some previous studies suggests that psychopathology and functional impairment share many risk or associated factors as was suggested by the current study. Factors found as significant risk factors of diagnosable psychopathology (younger age, chronic physical illness, poor academic performance, frequent hospital visits, parenting distress and maternal psychological morbidity) are consistent with some of those in some previous studies [28,31]. Many of these factors were equally significantly associated with functional impairment in addition to the history of developmental delays and poor spousal support. The similarity in factors associated with psychopathology and functional impairment is consistent with findings of a previous study conducted in a setting similar to ours [32]. Understanding the nature of risk factors of psychopathology and factors associated with functional impairment and how they interact is important in planning intervention strategies.

The significant association between psychopathology and functional impairment means that the pediatric clinic would have children with varying degree and isolated cases of both. On this basis and of similarities of risk factors all children should be assessed for evidence of both problems. It is advisable that children with both should be referred to mental health professional because they have a greater burden of morbidity [3].

Child and parental factors identified as predictors of diagnosable psychopathology in the present study (younger age, frequent hospital visits and parenting difficulty) are known risk factors [11,12,33] of child psychopathology our study has only confirmed and extended this evidence. Previous studies in primary care have suggested that children who are frequent hospital attendees have elevated risk of psychiatric morbidity consistent with our finding. Undetected psychological problems may contribute to avoidable hospital visits while chronic physical disorders may be a source of frequent obligatory visits and may also be an independent risk factor of child psychopathology [10] as suggested by this study. Chronic physical illness and its accompanying psychosocial problems adversely affect parent–child interaction patterns making both of them vulnerable to psychological dysfunction [10,30,31]. Parenting difficulty is another indicator of problems with parent–child interaction that predicted psychopathology in our study and is supported by some previous studies as an important factor in this regard [12,31,33]. Parenting skill enhancement programs should be part of psychosocial intervention in children with psychological problems as it may assist in preventing some of these problems. Generally, most parenting training in Nigeria is obtained via religious and cultural instructions. Clinic-based or formal parenting education, skill development and support programs whether group or individual or self-administered are rare in Nigeria to the best of our knowledge. On the basis of the present evidence mental health providers can collaborate with primary care providers to offer some parenting educational and support services during routine clinics pending the development of more specialized services.

One explanation for maternal psychiatric morbidity as a risk factor for child psychiatric morbidity is that it may also negatively affect the parent–child interaction and make the home environment unfavorable for adequate psychosocial adjustment and development, thereby increasing vulnerability to dysfunction and psychopathology in the children [31,33]. Finally, we noticed that many of the children with inappropriate educational status had chronic physical illness which could have affected them in this regard. The need for educational intervention as part of psychosocial rehabilitation of children is the implication of this finding. This will ensure that those with psychopathology are not educationally disadvantaged too.

Improving service provision by primary care physicians in our hospital in terms of detection, treatment and referrals was the focus of this study. Developing a protocol to assist in this regard should be the next line of action following the evidence that risk factors and functional impairments of child psychopathology are prevalent among children in our primary care unit. The mhGAP guide [34] will be of immense benefit in this regard because it is a protocol-based tool designed for low resource non-specialist setting like ours. It is brief, easy to use by busy non-specialist to deliver pharmacologic, non-pharmacologic and psychosocial child psychiatry care and referrals [34]. Our child and adolescent psychiatric unit will however need to provide necessary modifications to the tool, and training, support and supervision in line with principles of consultation and liaison psychiatry. The child psychiatric unit will also need to carry out more sensitization program targeted at primary care providers, teachers, parents and the community at large to further improve detection, treatment and referrals.

The numbers of children in the subcategory of disorders except for enuresis were too few for any detailed analysis. We therefore could not calculate risk levels for individual diagnostic subgroups; since this was not a primary research outcome objective it cannot be considered a limitation of serious importance. Causal relations could not be attached to the risk factors since this study was not a randomized controlled investigation. We should be more interested in children who had psychiatric diagnosis but were not dysfunctional. We did not explore the coping strategies of the children and how they were able to adjust; these are issues that future researches need to look at.

## Conclusions

Child psychiatric disorders are prevalent in primary care and undetected, and many of the affected children may be functionally impaired. Interrupting impairment at the primary care level is an important preventive strategy. Identification of risk factors of functional impairment and psychiatric morbidity in children would assist in formulating preventive strategies.

Primary care physicians and workers can improve their competence in case detection when they have the knowledge of local risk factors especially modifiable ones. For example, the fact that our study identified frequent hospital attendees to be at increased risk of psychiatric morbidity means that our primary care physicians should ask more psychiatry related questions when they encounter such patients.

Collaboration between psychiatrists and primary care physicians need to be encouraged in order to improve mental health service delivery, both at the primary care level and at the child and adolescent psychiatric facilities.

## Abbreviations

CBQ: Child Behavior Questionnaire; DSM IV: Diagnostic and Statistical Manual IV; CGAS: Children Global Assessment Scale; ADHD: Attention Deficit Hyperactivity Disorder; GOPD: General Outpatient Department; UITH: University of Ilorin Teaching Hospital; GHQ: General Health Questionnaire; K-SADS-PL: Schedule for Affective Disorders and Schizophrenia, Present and Life version; K-SADS: Schedule for Affective Disorders and Schizophrenia; EPI Info: Statistical Software for Epidemiology; SPSS: Statistical Package for Social Sciences.

## Competing interest

All authors declare no competing interest.

## Authors’ contributions

MF: Contributed to design, data acquisition, analysis and interpretation of data; drafted the manuscript and revised it critically for important intellectual content; and have given final approval of the version to be published. OA: Contributed to data acquisition, analysis and interpretation; and drafting of the manuscript; and have given final approval of the version to be published. BA: Contributed to data acquisition, analysed and interpreted the data; involved in drafting and revising the manuscript; and have given final approval of the version to be published. OAA: Contributed to conception and design, data acquisition, revised the manuscript critically for important intellectual content; and have given final approval of the version to be published.
